# Practice of preventive dentistry for nursing staff in primary care

**Published:** 2014-09-30

**Authors:** María Valeria Jiménez-Báez, Raquel Acuña-Reyes, Didier Cigarroa-Martínez, Enrique Ureña-Bogarín, Jose David Orgaz-Fernández

**Affiliations:** 1 Coordinación Auxiliar de Investigación en Salud. Jefatura de Prestaciones Médicas. IMSS. Cancún Quintana Roo . México; 2 Estomatólogía. Cancún Quintana Roo. México; 3 Estomatología UMF no. 14. IMSS. Cancún Quintana Roo. México; 4 Coordinación de Planeación y Enlace Institucional. Jefatura de Prestaciones Médicas. IMSS. Veracruz Sur. México; 5 Jefe de Prestaciones Médicas. IMSS. Cancún Quintana Roo. México

**Keywords:** Preventive dentistry, level of knowledge, nurses

## Abstract

**Objectives:**

Determine the domain of preventive dentistry in nursing personnel assigned to a primary care unit.

**Methods:**

Prospective descriptive study, questionnaire validation, and prevalence study. In the first stage, the questionnaire for the practice of preventive dentistry (CPEP, for the term in Spanish) was validated; consistency and reliability were measured by Cronbach's alpha, Pearson's correlation, factor analysis with intra-class correlation coefficient (ICC). In the second stage, the domain in preventive dental nurses was explored.

**Results:**

The overall internal consistency of CPEP is α= 0.66, ICC= 0.64, CI95%: 0.29-0.87 (*p* >0.01). Twenty-one subjects in the study, average age 43, 81.0% female, average seniority of 12.5 were included. A total of 71.5% showed weak domain, 28.5% regular domain, and there was no questionnaire with good domain result. The older the subjects were, the smaller the domain; female nurses showed greater mastery of preventive dentistry (29%, CI95%: 0.1-15.1) than male nurses. Public health nurses showed greater mastery with respect to other categories (50%, CI95%: 0.56-2.8).

**Conclusions:**

The CDEP has enough consistency to explore the domain of preventive dentistry in health-care staff. The domain of preventive dentistry in primary care nursing is poor, required to strengthen to provide education in preventive dentistry to the insured population.

## Abstract (es)

**Objetivos:**

Determinar el dominio sobre estomatología preventiva en personal de enfermería adscrito a una unidad de atención primaria.

**Métodos:**

Estudio descriptivo prospectivo, validación de cuestionario y estudio de prevalencia. En la primera etapa, se validó el cuestionario para la práctica de estomatología preventiva (CPEP), se midió consistencia y fiabilidad mediante Alpha de Cronbach, correlación de Pearson, análisis factorial con el coeficiente de correlación intraclase (CCI). En la segunda etapa, se exploró el dominio de Estomatología preventiva en enfermeras.

**Resultados:**

La consistencia interna global del CPEP es α= 0.66, CCI= 0.64 IC95%: 0.29-0.87 (*p*> 0.01). Se incluyeron 21 sujetos en el estudio, promedio de edad 43 años, 81.0% del sexo femenino, antigüedad laboral media de 12.5. El 71.5% con dominio malo, 28.5% regular y no existió ningún cuestionario con resultado bueno. A mayor edad menor dominio; las enfermeras mostraron un dominio mayor de estomatología preventiva (29%, IC95%: 0.1-15.1) que los enfermeros. Enfermeras especialistas en salud pública mostraron un dominio mayor con respecto a las otras categorías (50%, IC95%: 0.56-2.8).

**Conclusiones:**

El CDEP tiene une consistencia suficiente para explorar el dominio de estomatología preventiva en personal del área de la salud. El dominio sobre estomatología preventiva en enfermería de atención primaria es malo; se precisa fortalecer para otorgar educación en estomatología preventiva a la población derechohabiente.

## Introduction

Worldwide, the most common oral diseases in the oral cavity are caries and periodontal diseases 1 ^-^ 3, with children 4 ^,^ 5, pregnant women 6, and older adults 7 ^-^ 8 being the most vulnerable groups 9. Oral diseases are a reflection of the health status of the population; the functions the stomatological device plays, besides being different, are essential to a good quality of life 10.

Environmental features such as reduced exposure to topical fluoride 11 ^-^ 13, poor resistance of enamel, dental crowding, foods containing simple sugars between meals, malnutrition 5 ^,^ 10 ^,^ 14, low socioeconomic status, unusual visits to the dentist, caries activity in the mouth increases the risk of developing caries. While health conditions that increase the risk of caries are the special needs of the patient and the composition, viscosity, and pH of salivary flow 6 ^,^ 13 ^-^ 15.

Dental caries is considered by the World Health Organization (WHO) the third most-common systemic disease in several emerging countries, including Mexico 1 ^,^ 15 ^,^ 16, 90% of the population in the country has caries and between 40 and 90% of the children have them; it is one of today's most costly diseases, given that dental treatments account for 5 to 10% of health spending that is beyond the resources of many developing countries and has a lifetime duration 11 ^,^ 17.

Periodontal disease is the second most-common oral disease in the world population, WHO reveals that 60% of the population suffers from this pathology and that no country in the world or territory is free of said disease; besides, these originate more teeth loss due to tooth decay 1. In Mexico, it affects between 50 and 60% of the population 16 ^,^ 17.

The dental prevention strategy has shown to improve the oral health status of the Mexican population 18; proper education that the staff gives to the patient is a critical pillar to achieving impact in reducing diseases related to poor oral health. The Mexican Social Security Institute (IMSS, for the term in Spanish), through the process of continuous improvement, implemented in 2002 the strategy of integrated health programs known as PREVENIMSS, which is updated every year and refers to preventive actions that improve the health of its beneficiaries; it includes a strategic line of preventive dentistry aimed at different age groups within the population 19 ^,^ 20.

It is imperative for nurses to know: 1. The purpose of preventing dental disease. 2. The most common oral pathologies. 3. Common techniques to identify and remove plaque. 4. The specific function and correct application of topical fluoride 18 ^-^ 20.

Most studies currently published on the level of knowledge on preventive stomatology refer to patients 8 ^,^ 21 ^,^ 22. In the area of ​​health-care staff knowledge on oral health, little has been explored. In a study of 18 caregivers, whose purpose was to evaluate the profile and awareness of oral health, conducted in the city of Botocatu in Brazil by Saliba NA; 83.3% of them had a technical nursing assistant course; lack of information was detected in most of them, 55.5% believe that tooth loss is part of the aging process 8. In another study of Family Physicians by Muñoz-Muñiz in 2006, the level of knowledge on preventive dentistry was evaluated, 40% of the participants obtained a level of fair to good, which is insufficient 22.

The results presented in these studies show that the knowledge of health-care personnel is insufficient and inadequate; hence, if this group does not have precise knowledge on oral health prevention of oral diseases in the population may not be achieved. Dental caries and periodontal disease are the two most common diseases in the oral cavity; preventing such is simple through information about the cause and development 23 ^-^ 26. Prior to consulting with the dentist, patients have the first contact with nurses in preventive medicine and to whom they sometimes express doubts about the oral health of patients who may receive valuable information 18.

In order to determine the level of knowledge on preventive dentistry with nurses involved in the primary care unit of this study, the following validation of an instrument to that effect is made.

## Material and Methods

### Design and field of study

A cross-sectional descriptive study to validate an instrument for exploring the domain of preventive dentistry for primary care nurses was conducted. Randomly, a primary care unit of a total of 8 was selected in Quintana Roo, Family Medicine Unit No. 14, located in the northern region of the state in Cancún, Quintana Roo, in the year 2010 was selected. The sample consisted of nursing staff. 

### Participants

The participants were included according to the phase of the study. The content validation phase involved five experts in the field. The validity construction phase randomly selected an unrelated group (13 high-school students). The dentistry practice phase consecutively selected nurses at work and these were provided the self-managed CPEP, along with a sheet of demographic data.

### Measurements

**Figure 1.**
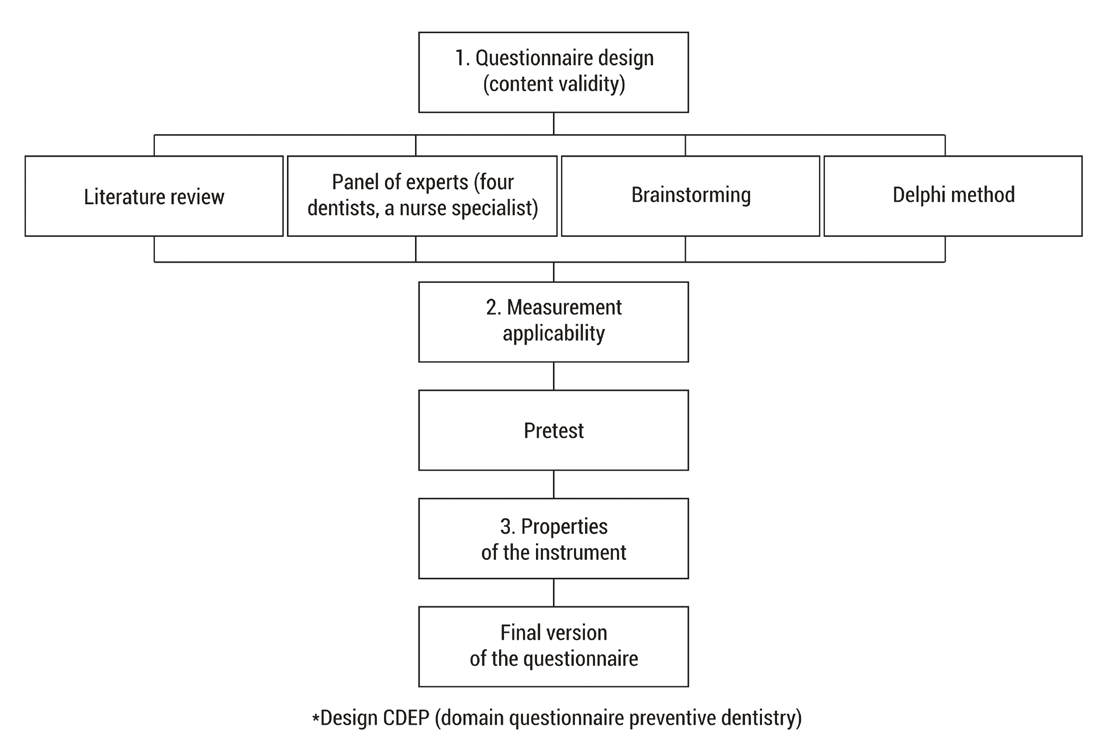
Flow diagram to questionnaire validation.

The study was conducted in three phases: the first phase, involving CPEP content validity, was considered by expert consensus and carried out the questionnaire's preliminary design based on existing theoretical assumptions in previous studies related to the domain of health-care personnel in preventive dentistry 24 ^,^ 25 and on the basis of pre-existing critical and constructive scales in doctors 22, caregivers 8 or nurses. It was then proceeded to elaborate the CPEP (Fig. 1) for analysis by a panel of experts integrated by four dentists and a specialist nurse, all of them with at least 4 years of experience in order to obtain a concordance index calculating Kappa (K= 1). Four clinical dimensions were considered: 1) Frequent mouth diseases and risk factors, items 1, 2, 4, 7, 8, 20. 2) Teething, items 3, 5, 6, 19. 3) Most-common techniques to identify and remove plaque, items 9, 11, 13, 15, 16, 21. 4) Specific function and correct application of topical fluoride, items 10, 12, 14, 17, 18, 20. Twenty-items with closed-type questions and one item with an open-type question. The overall results of the questionnaire were *good *with 17 to 21 items correct; *average* with 12 to 16 items and *poor* with less than 11 items.

In phase two, the construct validity was determined by applying the questionnaire to groups of unrelated people. Validation of the final CPEP version was made by analyzing its reliability and validity. The reliability was validated by measuring the internal consistency and homogeneity of the CPEP items, using the Cronbach's alpha values ​​of 0-1. We considered the minimum acceptable level of 0.6 and the reproducibility test-retest was performed on two different occasions separated by a one week interval. Analyzing both tests, with the variance test for independent samples (ANOVA). The factorial analysis was obtained through the following statistical test: intraclass correlation coefficients for repeated measures with decomposition in different sources (intra-subject, inter-subject, error and total) and the Pearson correlation coefficient (*r*).

Phase three: sample size was gotten out of convenience, unit's nursing staff was included if they had the criteria for participation in the study, to have permanent or temporary appointment as a nurse in the family medicine unit, those in vacation or disability periods were excluded, incomplete questionnaires were eliminated. Twenty-one nurses were included, which allowed estimating the parameters of interest with 95% (α= 0.05) and accuracy of ±5%. We proceeded to apply the CPEP and variables were recorded: age in years, education, highest level of education; category of procurement, service assigned to the unit, years of seniority, socioeconomic level.

### Statistical analysis

Quality control of the data was performed with the definition of the possible input values ​​for each field. The statistical analysis was performed by using SPSS^®^ version 20.0 for Windows^®^ 7.

### Ethical issues

Upon approval of the project by the local research committee, Registration No. R2008-2301-14, all participants were requested to sign informed consent to participate in the study. The study followed ethical rules and guaranteed the confidentiality of the data.

## Results

### Consistency 

The value of Cronbach's alpha for the entire questionnaire is 0.66. Table 1 shows the value obtained in any of the items, and the analysis of reproducibility by test re-test (n= 13), the correlation among the overall scores to determine the intra-class correlation coefficient (ICC) was equal to 0.64, CI95%: 0.29-0.87 (*p* >0.01).

**Table 1.**
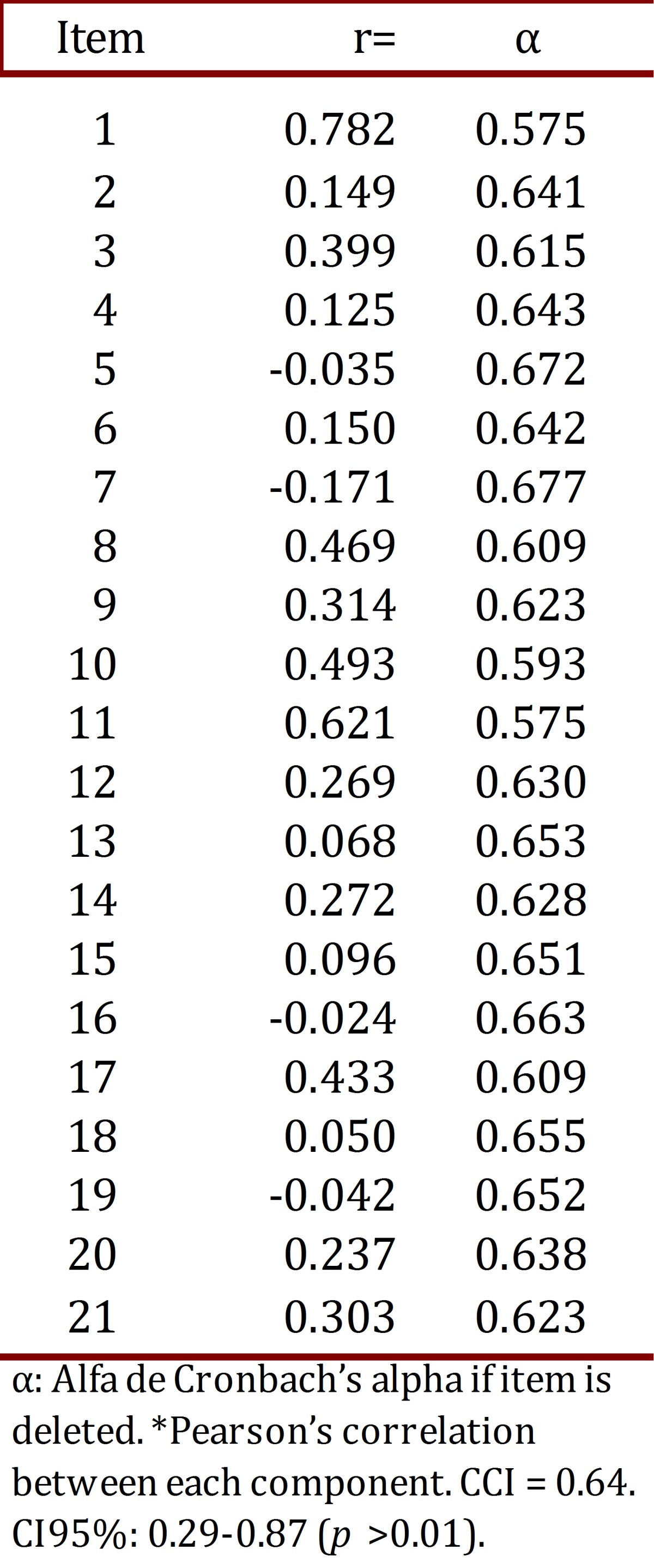
Test of consistency in each item of the CDEP.

### Validity

The mean scores of the items included in each dimension of CPEP were compared by the Friedman ANOVA test (Table 2).

**Table 2.**
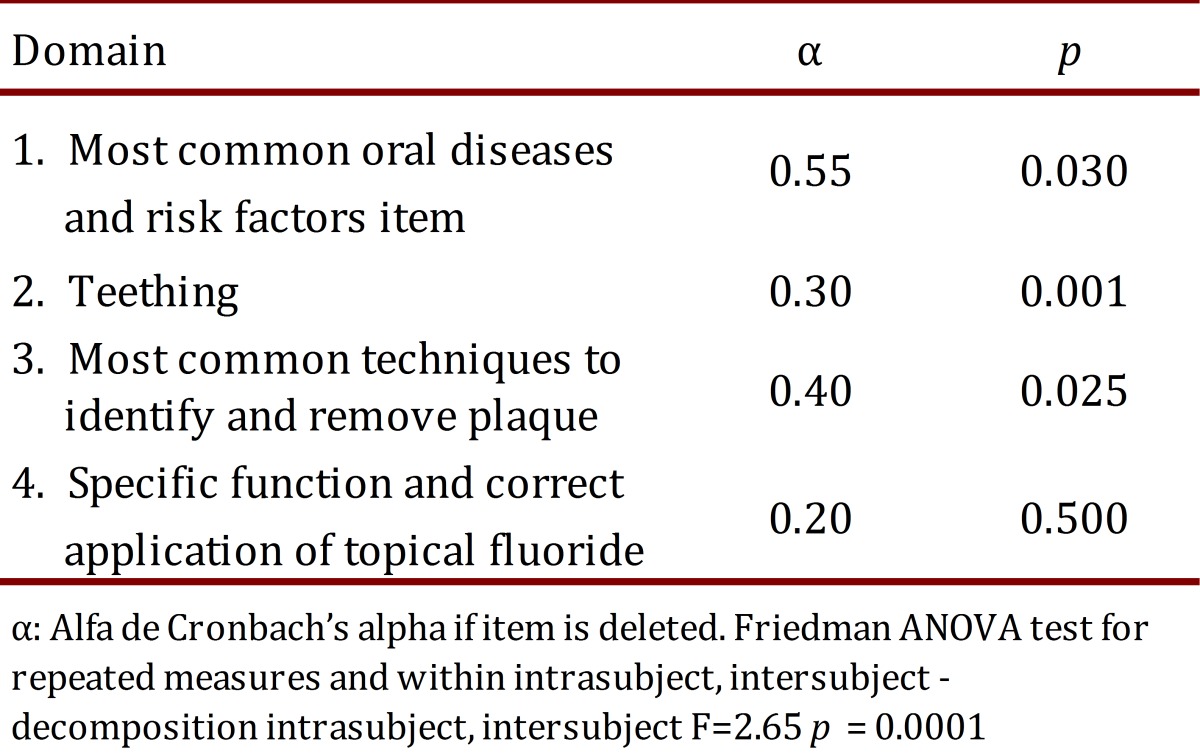
Consistency test in each domain in the CDEP. Global consistency α= 0.66.

### Application in primary care unit

The study surveyed 21 nurses assigned to a primary care unit of IMSS in Cancun - Quintana Roo, 81% (n= 17) of the population were female and 19% (n= 4) were male. The age range from 34-52 years, with a mean age of 43 years; the age range from 40 to 44 years had the highest percentage of this age group with 66.7% (n= 11). Regarding schooling, 15 (71.4%) subjects with upper secondary education; the socioeconomic level of the study population was predominantly medium high with 52.4% (n= 11), 10 were auxiliary nurses (47%), old working range was one to 24 years old, with a mean of 12.5 years.

The overall rating of the questionnaire was poor to average with a 71.5% (n= 15) of cases; whereas, 28.5% (n= 6) was average and, there were no questionnaires good results.

Females made up the majority of the population with a score of 29.4% (n= 12) in regular knowledge, furthermore there were no significant differences between sexes (*p* >0.05). Staff between the ages of 40 and 44 had the highest percentage of regular results with 36.4% (n= 4), no correlation was found between age and knowledge levels r= 0.2 (*p *>0.05). Nurses belonging to the medium-lower socioeconomic level obtained an average rate of 33.3% (n= 3).

According to schooling, subjects with a higher level of education obtained 26.6% (n= 4) of average results; no significant differences according to the education level (*p* >0.05) and employment status were found. Nurses in Public Health showed a 50.0% (n= 2) average score of knowledge in preventive dentistry. Nurses with 10 to 14 years in work achieved an average score of 50.0% (n= 2) as did subjects with 15 to 19 years. Seniority of staff in the preventive medicine service were 5 years 83.3% (n= 5) and 3 years in 16.8% (n= 1).

## Discussion

Instruments used to measure the domain of preventive dentistry by nurses in primary care units are not routinely used. Recently published studies are focused on investigating patient knowledge 8 ^,^ 21 ^,^ 22 ^,^ 26 ^-^ 28, there is no *gold standard*, our obtained results by context validation, construct and applicability give us an option with enough consistency to explore this area of practice in primary care units.

The results presented in these studies show that the knowledge of health-care staff 8 ^,^ 21 ^,^ 22 is insufficient and inadequate; thereby, if this group does not have precise knowledge on oral health prevention of oral diseases cannot be achieved in the population.

Knowledge in preventive stomatology of the most-common diseases affecting patients of primary care users such as caries and periodontal diseases 1 ^,^ 3 ^,^ 4 allow us to train and evaluate the impact on those patients who influence in the rate decrease of these preventable diseases 29 ^-^ 31. Saliba *et al.,* found results similar to ours investigating the older caregivers profile and their oral health perception where the knowledge level was poor in the majority of participants 8, while in the study applied in physicians by Muñoz-Muñiz regular to good results were observed 22, which might suggest that family physicians have a bigger knowledge about preventive stomatology than the nursery personnel in spite of official norm dictating that all the health area work personal should have the basic knowledge in this area 18.

The comparison between knowledge levels and observed results for the population's features like sex, age, education, socioeconomic status, category and labor years were treated with the *X* *2* test although the existence of clinical significance without statistical significance, (*p *<0.5) allowing us to suggest the realization of larger subject number studies. There is an area of ​​opportunity in implementing educational programs on preventive dentistry, resulting in the improvement of staff skills.

The lack of knowledge for those who are an important part in the leading of this major oral diseases prevention programs represents a barrier to preventing these diseases.

The weakness of this study was the number of questionnaires completed (n= 21) relative to the number of nurses (n= 29) either because it was a holiday period or they chose not to participate in the study, which did not allow us to study the entire nursing population.

The study's strength was manifested by the lack of preventive stomatology studies in health workers; in spite of an exhaustive bibliographic search not many research studies in this area have been made. Based on this, it allowed an opportunity for the implementation to improve strategies in the unit whose yield will be an entitled attention quality improvement generating a favorable outcome aimed to mobility decrease.

As the first contact, nurses can strengthen periodontal disease prevention and reduce frequency. A positive effect is an appropriate knowledge about preventive measures is the reduction of oral disease incidence. First contact population in primary care units requires a consistent education in oral diseases prevention.

The level of knowledge of preventive dentistry in nursing staff from a primary care unit is predominantly poor, although public international policies are focused on strengthening oral health 1 ^,^ 18, care processes on first contact whose task is to implement these policies 19 ^,^ 20 is unrelated to the absence of a solid knowledge to impact oral health prevention practices of patients.

An option for health providers to prevent major oral diseases is the use of instruments focused on specific oral preventive actions and aligned with international policies.
